# Blood and Salivary Amphiregulin Levels as Biomarkers for Asthma

**DOI:** 10.3389/fmed.2020.561866

**Published:** 2020-10-29

**Authors:** Mahmood Yaseen Hachim, Noha Mousaad Elemam, Rakhee K. Ramakrishnan, Laila Salameh, Ronald Olivenstein, Ibrahim Yaseen Hachim, Thenmozhi Venkatachalam, Bassam Mahboub, Saba Al Heialy, Rabih Halwani, Qutayba Hamid, Rifat Hamoudi

**Affiliations:** ^1^College of Medicine, Mohammed Bin Rashid University of Medicine and Health Sciences, Dubai, United Arab Emirates; ^2^Sharjah Institute for Medical Research, College of Medicine, University of Sharjah, Sharjah, United Arab Emirates; ^3^Rashid Hospital, Dubai Health Authority, Dubai, United Arab Emirates; ^4^Meakins-Christie Laboratories, McGill University, Montreal, QC, Canada; ^5^Department of Clinical Sciences, College of Medicine, University of Sharjah, Sharjah, United Arab Emirates; ^6^Division of Surgery and Interventional Science, UCL, London, United Kingdom

**Keywords:** asthma, biomarkers, saliva, amphiregulin (AREG), non-invasive

## Abstract

**Background:** Amphiregulin (AREG) expression in asthmatic airways and sputum was shown to increase and correlate with asthma. However, no studies were carried out to evaluate the AREG level in blood and saliva of asthmatic patients.

**Objective:** To measure circulating AREG mRNA and protein concentrations in blood, saliva, and bronchial biopsies samples from asthmatic patients.

**Methods:** Plasma and Saliva AREG protein concentrations were measured using ELISA while PBMCs, and Saliva mRNA expression was measured by RT qPCR in non-severe, and severe asthmatic patients compared to healthy controls. Primary asthmatic bronchial epithelial cells and fibroblasts were assessed for AREG mRNA expression and released soluble AREG in their conditioned media. Tissue expression of AREG was evaluated using immunohistochemistry of bronchial biopsies from asthmatic patients and healthy controls. Publicly available transcriptomic databases were explored for the global transcriptomic profile of bronchial epithelium, and PBMCs were explored for AREG expression in asthmatic vs. healthy controls.

**Results:** Asthmatic patients had higher AREG protein levels in blood and saliva compared to control subjects. Higher mRNA expression in saliva and primary bronchial epithelial cells plus higher AREG immunoreactivity in bronchial biopsies were also observed. Both blood and saliva AREG levels showed positive correlations with allergic rhinitis status, atopy status, eczema status, plasma periostin, neutrophilia, Montelukast sodium use, ACT score, FEV1, and FEV1/FVC. *In silico* analysis showed that severe asthmatic bronchial epithelium with high AREG gene expression is associated with higher neutrophils infiltration.

**Conclusion:** AREG levels measured in a minimally invasive blood sample and a non-invasive saliva sample are higher in non-allergic severe asthma.

**Clinical Implications:**

This is the first report to show the higher level of AREG levels in blood and saliva of non-allergic severe asthma.

## Introduction

Asthma is characterized by chronic airway inflammation, mucus hyper-production, airway hyper-responsiveness, and variable airway obstruction (1). The prevalence of asthma has increased in the last few years, reaching alarming levels (2). Understanding the complexity of asthma will help in establishing management strategies aiming for better asthma patients stratification for personalized therapeutic options (3). Current biomarkers include eosinophil counts, a fraction of exhaled nitric oxide (Feno) values, periostin, IgE levels, and T-helper2 related cytokines (4). The identification of novel, cost-effective, reliable, and measurable non-invasive markers (5) is the goal for many research projects (6–8).

The epidermal growth factor receptor (EGFR) signaling is vital to epithelial cell physiology, and its dysregulation is involved in different pathologies (9), including pathogenesis of early stage of asthma (10). Many asthma triggers like allergens, viruses, and pollutants activate EGFR signaling, which seems to be a common pathway shared by different asthma phenotypes (11). EGFR signaling mediates airway hyper-responsiveness (AHR), tissue remodeling (12) and is implicated in lung fibrosis (10). Amphiregulin (Areg), an EGFR ligand, and a widely expressed transmembrane tyrosine kinase (13, 14), was shown to be upregulated upon inflammation, hormones, growth factors, and xenobiotics stimuli (13). Previously, high AREG expression was related to lung inflammation (15), specifically in damaged lung tissues in patients with chronic obstructive pulmonary disease (COPD) and asthma (16). AREG expression in structural cells (15) and sputum was associated with asthma severity (17), and it was shown to be increased in airways during an acute asthma attack (18).

Interestingly, hematopoietic cells that infiltrate inflamed lung tissue were shown to locally upregulate AREG expression and influence its local concentrations (15). While human monocytes were found to express AREG upon activation (19), eosinophils still represent the primary producers of AREG when compared to other types of blood cells (19) and can be reprogrammed to an inflammatory state by AREG- EGFR-mediated signaling (20). For all the above reasons, AREG was suggested to be linked to severe eosinophilic asthma with less response to asthma therapy (21). Such sensitized individual develops eosinophilic airway inflammation after the exposure to allergens through IgE antibody-mediated mast cell degranulation is labeled as allergic asthmatic. On the other hand, a non-allergic asthmatic is a non-sensitized individual who can develop eosinophilic inflammation without exposure to an allergen through the activation of ILC2 cells that do not need allergen-specific IgE (22). ILC2s and lung activated alveolar macrophages can control lung inflammation by producing AREG to promote repair of the airway epithelium, that is why depletion of these cells can result in diminished lung function after the loss of airway epithelial integrity which can be restored by adding AREG (23–26).

Non-allergic patients may have innate immunity or neutrophilic airway inflammation independent of Th2 cells (27). Triggers of asthma in non-allergic patients can vary from external triggers like viral infections, cigarette smoke, diesel particles, and ozone to patient's intrinsic factors like stress, exercise, and obesity (1). Following epithelial damage, the epidermal growth factor (EGF) can enhance the neutrophil accumulation to stimulate neutrophil defenses during acute injury (28).

Of note, T cells in peripheral blood mononuclear cells (PBMC) can also produce AREG in response to signals of tissue damage and repair (29). The association of a protective AREG with disease progression, and whether there is cell to cell variation in production and response to AREG remain unknown.

Some circulating plasma proteins originating from tissue leakage (30) and thus being detected in saliva and can reflect their upregulation in serum/plasma (31). Because saliva is easy to collect, innocuous, acceptable by patients, thus it represents a potential tool for measuring disease biomarkers (32). Therefore, measuring the expression of AREG in plasma and saliva from asthmatic patients can provide a minimally invasive approach for the assessment of AREG, which may provide a more reliable tool complementary to existing measures.

## Methods

### Patient Population (Cohort 1)

From January 2017 to May 2019, individuals were recruited consecutively from the Asthma Clinic in Rashid Hospital, Pulmonary Medicine Department. Thirty-two asthmatic patients were included, 19 were non-severe asthmatic patients (mild to moderate), and 13 were severe asthmatic subjects (fulfilling the criteria for asthma as per American Thoracic Society) (Table 1). According to GINA and ERS/ATS Task Force on severe asthma, guidelines define asthma severity as “those who require continuous high-dose treatment, as their asthma is refractory or difficult-to-treat plus a second controller (and/or systemic corticosteroids) to prevent it from becoming uncontrolled or which remains uncontrolled despite this therapy” (33). Those patients were compared to 12 non-asthmatic volunteer subjects who had no recent infection of the respiratory tract and no histories of allergy or asthma. Participants completed the Asthma Control Test self-assessment. The Ethics Committee of Dubai Health Authority and the University of Sharjah approved the study with REC (Research Ethics Committee) approval number DSREC-11/2017_04, and each subject gave written informed consent after a thorough explanation by the treating physician and the researchers. This study was conducted in accordance with the Declaration of Helsinki. The demographic characteristics of the asthmatic patients and control subjects are shown in **Table 4**.

**Table 1:** Characteristics of the cohort 1 subjects with asthma and healthy controls.

	**Control**	**Non-severe asthma**	**Severe asthma**	***P*-value**
Number of subjects	12	19	13.00	
Age (Years)	37.00 ± 11.73	40.19 ± 15.65	48.42 ± 17.95	NS
Age of onset (adult to childhood ratio)				
Childhood asthma		11	5	
Adult asthma		5	8	
Female to male ratio	8:4	13:3	7:6	NS
BMI	24.60 ± 3.19	27.63 ± 6.40	27.03 ± 6.56	NS
ACT score	–	20.75 ± 4.31	17.55 ± 6.15	NS
Oral steroid use per day/week	–	0.6563 ± 1.06	2.083 ± 1.459	0.01
Exacerbations/year	–	1.688 ± 2.575	2.133 ± 1.482	NS
History of atopy				
Yes	0	0	4	<0.01
No	10	16	9	
History of allergic rhinitis				
Yes	1	10	9	<0.01
No	9	6	4	
Peak flow (l/min)	453.80 ± 101.00	299.30 ± 78.20	302.50 ± 139.90	<0.01
FEV1 (% predicted)	–	55.46 ± 42.42	54.06 ± 45.53	NS
FEV1/FVC	–	56.87 ± 40.30	49.13 ± 35.35	NS
Total serum IgE (IU/mL)	–	280.30 ± 358.40	1167.00 ± 1428.00	<0.01
Blood eosinophil (%)	–	4.54 ± 3.24	6.88 ± 9.57	NS
Blood neutrophil (%)	–	58.29 ± 12.34	60.04 ± 13.86	NS
Blood basophil (%)	–	0.53 ± 0.36	0.52 ± 0.24	NS
Blood lymphocytes (%)	–	28.89 ± 11.05	26.67 ± 12.15	NS
Absolute eosinophil counts (cells/μL)	–	181.6 ± 129.6	275.2 ± 382.8	NS

### Patient Population (Cohort 2)

A matched cohort of severe asthmatics (*n* = 17) vs. non-severe asthmatics (*n* = 21) was recruited for fresh saliva collections and RNA extractions was performed to explore the feasibility of detecting AREG mRNA in the saliva.

### Blood Collection Protocol and Plasma Isolation

Twelve milliliters of whole blood were collected from each sample in EDTA-containing blood collection tubes (3 mL each) and transferred immediately within 2 h to Sharjah Institute for Medical Research (SIMR), Sharjah for further processing to isolate PBMCs as previously described (34). Twelve milliliters of Histopaque-1077 (Sigma, #10771, Germany) were added to a 50 mL centrifuge tube and brought to room temperature (RT), then 12 mL of whole blood were carefully layered on top of the Histopaque and centrifuged at 400 × g for precisely 30 min at room temperature. After centrifugation, the plasma layer and the buffy layer interface were carefully collected with separate Pasteur pipettes and transferred to clean 15 mL conical centrifuge tubes separately, to be frozen at −80°C till future use.

### Unstimulated Whole Saliva Collection Protocol

Participants were asked to fast for at least 1 h and not to brush their teeth or smoke for 30 min, accompanied by gargling and rinsing of the mouth with water 5 min before proceeding with saliva collection. One milliliter of unstimulated whole saliva via passive drool was collected in a pre-prepared 50 mL tube containing 1 mL of RNAlater (Invitrogen). Collected saliva samples were transported on ice and stored at – 80°C until analysis. Sample measurements were undertaken within 3 months of storage.

### Primary Cell Lines and Bronchial Biopsies (Cohort 3)

Primary cells from healthy and asthmatic patients were isolated from bronchial biopsies in Meakins-Christie Laboratories, The Centre for Respiratory Research at McGill University and the Research Institute of McGill University Health Centre as previously described (35). In total, 17 primary cells were exploited: 7 healthy primary cells: epithelial (*n* = 3) and fibroblasts (*n* = 4), and 10 primary asthmatic cells: epithelial cells (*n* = 3), non-severe asthmatic fibroblasts (*n* = 4), and severe asthmatic fibroblasts (*n* = 3). Twelve bronchial biopsies were taken from control (*n* = 3) and asthmatic (*n* = 9, 3 in each clinical stage: mild, moderate, and severe) were used for immunohistochemical assessment. Epithelial cells were revived and maintained using epithelial growth medium PneumaCult™-Ex Medium (Stem Cell Technology, Canada), supplemented with 100 units/mL penicillin/streptomycin (Gibco, USA). Primary fibroblasts were maintained in complete Dulbecco's Modified Eagle's medium (DMEM) (Sigma-Aldrich, Germany) with 10% fetal bovine serum (FBS) (Sigma-Aldrich, Germany) supplemented with 100 units/mL penicillin/streptomycin (Gibco, USA).

### mRNA Gene Expression Using qRT-PCR

RNA was extracted using RNAeasy mini kit (Qiagen, Germany) as per the manufacturer instructions. The purified RNA was reverse transcribed into cDNA using High Capacity cDNA Reverse Transcription (Applied Biosystems, USA) as per the manufacturer instructions. 5X Hot FIREPol EvaGreen qPCR Supermix (Solis BioDyne, Estonia) was used to quantify mRNA of the selected genes using QuantStudio3 (Applied Biosystems, USA). Details of used primers are in Table 2.

**Table 2:** List of forward and reverse primers for each of the genes assessed by qRT-PCR.

**Gene**	**NCBI reference sequence**	**Primer**	**Sequence (5^**′**^-3^**′**^)**	**Amplicon size (bp)**
Areg	NM_001657.4	Forward primer	GAGCACCTGGAAGCAGTAAC	151
		Reverse primer	GGATCACAGCAGACATAAAGGC	
18S rRNA	NR_145820.1	Forward primer	TGACTCAACACGGGAAACC	114
		Reverse primer	TGCCTCCACCAACTAAGAAC	

### Enzyme-Linked Immunoassay (ELISA) Quantification

Human amphiregulin was measured in plasma and saliva of healthy controls and asthmatic patients as well as the conditioned media of primary bronchial cells using the Human Amphiregulin Quantikine and Duoset ELISA Kits (R&D Systems, USA) according to the manufacturer's instructions. Periostin (POSTN) and IL-17A were assessed in plasma using human Periostin/OSF-2 and human IL-17A DuoSet ELISA Kits (R&D Systems, USA), respectively, according to the manufacturer's instructions.

### Soluble Receptors Array

To assess any difference in the shedding of AREG in the conditioned media of the asthmatics vs. healthy primary fibroblasts, Non-Hematopoietic Array and the Common Analytes Array (R&D Systems, Catalog # ARY011) was used as per the manufacturer instructions. The conditioned media of healthy bronchial fibroblasts and non-severe asthmatic fibroblasts were collected, centrifuged and 1.5 mL of each were added to the array wells separately and incubated overnight on a rocking shaker. On the next day, the membranes were washed to remove any unbound material and incubated with the specific cocktail of biotinylated detection antibodies. Streptavidin-horseradish peroxidase and chemiluminescent detection reagents were added, and a chemiluminescent signal was produced in proportion to the amount of receptor-bound. Dot blots were registered with ChemiDoc Imaging System (BioRad, USA). The average pixel density of duplicate spots on the membrane was determined using ImageJ software (36). After background subtraction, the relative amounts of individual proteins were calculated as previously reported (37).

### Immunohistochemical Assessment of Bronchial Biopsies

Immunohistochemical evaluation of bronchial biopsies from healthy and asthmatic patients was done on a 3 μm section of the tissue block mounted on positively charged slides. After routine deparaffination and rehydration, antigen retrieval using Tris EDTA buffer (pH 9.0) in a microwave oven at 95°C for 15 min was done. Horse radish peroxidase (HRP) kit (760-700, Optiview DAB IHC Detection kit, Ventana) was used as per the manufacturer instructions, along with neutralizing antibody for AREG (15 μg/mL) (R&D Systems, USA) for AREG detection.

### *In silico* Validation in Lung and Blood Transcriptomic Datasets

Twenty-five publicly available transcriptomics datasets (*n* = 2,666) of Bronchial Epithelium, Bronchial Fibroblasts, Whole Lung, Bronchial Biopsy, Induced Sputum, Nasal, Bronchioalveolar lavage, Blood, Whole Blood, PBMCs, and CD4 cells from asthmatic patients vs. healthy subjects in different settings were downloaded from Gene Expression Omnibus (https://www.ncbi.nlm.nih.gov/geo). The raw data was extracted according to Hachim et al. (38) and the normalized gene expression of the AREG was compared between patients with different asthma severities as shown in Table 3.

**Table 3:** List of datasets extracted from GEO omnibus for the in silico analysis.

**Dataset ID**	**Tissue/sample**	**Study title**	**Samples**
GSE4302	Bronchial epithelium	Genome-Wide Profiling of Airway Epithelial Cells in Asthmatics, Smokers and Healthy Controls	70
GSE18965	Bronchial epithelium	Decreased Fibronectin Production Significantly Contributes to Dysregulated Repair of Asthmatic Epithelium	16
GSE43696	Bronchial epithelium	Asthma	108
GSE51392	Bronchial epithelium	Expression Data From Airway Epithelial Cells Stimulated With Poly(I:C) From Patients With Asthma, Rhinitis, and Healthy Controls	12
GSE67472	Bronchial epithelium	Airway Epithelial Gene Expression in Asthma Versus Healthy Controls	105
GSE64913	Bronchial epithelium	Altered Epithelial Gene Expression in Peripheral Airways of Severe Asthma	36
GSE76227	Bronchial epithelium	Expression Data of Bronchial Biopsies and Epithelial Brushing From Unbiased Biomarkers in Prediction of Respiratory Disease Outcomes (U-BIOPRED) Project	190
GSE13785	Bronchial epithelium	Novel Mediators of Eicosanoid and Epithelial Nitric Oxide Production in Asthma	22
GSE30063	Bronchial epithelium	Epithelial Expression of Toll-like Receptor 5 is Modulated in Healthy Smokers and Smokers With Chronic Obstructive Lung Disease	63
GSE27335	Bronchial fibroblasts	Genomic Differences Distinguish the Myofibroblast Phenotype of the Distal Lung From Airway Fibroblasts	24
GSE21369	Whole lung	Gene Expression Profiles of Interstitial Lung Disease (ILD) Patients	6
GSE41649	Bronchial biopsy	Comparison of Two Sets of Microarray Experiments to Define Allergic Asthma Expression Pattern	8
GSE56396	Induced sputum	Non-invasive Analysis of the Airway Transcriptome Discriminates Clinical Phenotypes of Asthma	112
GSE41863	Induced sputum	Sputum Gene Expression Profiling in Asthmatics	56
GSE41861	Nasal	Upper Airway Gene Expression Is an Effective Surrogate Biomarker for Th2-Driven Inflammation in the Lower Airway	84
GSE41862	Nasal	Nasal Scrape Gene Expression Profiling in Asthmatics	116
GSE67940	Bronchioalveolar lavage	Asthma III	104
GSE115823	Blood	A Network of Transcriptome Modules Demonstrates Mechanistic Pathways of Both Virus-Induced and Non-viral Asthma Exacerbations in Children [Blood]	208
GSE69683	Blood	Expression Profiling in Blood From Subjects With Severe Asthma, Moderate Asthma, and Non-asthmatics Collected in the U-BIOPRED Study	498
GSE35571	Blood	Gene Expression Data From 131 Human Subjects in Detroit, Michigan	131
GSE137394	Whole blood	Genome-Wide Profiling of Allergic Asthma Peripheral Blood	309
GSE123750	Whole blood	U-BIOPRED Blood Transcriptomics From children With Asthma or Wheeze	216
GSE31773	PBMC	Comparison of mRNA Expression in Circulating T-Cells From Patients With Severe Asthma	40
GSE16032	PBMC	Gene Expression Data From Severe Asthmatic Children: PBMC Profiles During Acute Exacerbation Versus Convalescence	10
GSE73482	CD4	Gene Expression Patterns in Allergen-Driven CD4 T Cell Responses From Human Atopics With or Without Asthma	144
Total	2,666		

### *In silico* Prediction of the Percentage and Status of Immune Cells

We used the transcriptomic data to predict the percentage and status of immune cells in the bronchial epithelium or blood of severe asthmatics patients compared to the healthy controls using CIBERSORT computational method (https://cibersort.stanford.edu/) to quantify cell fractions from samples gene expression profiles as previously described (39).

### Statistical Methods

GraphPad Prism version 8.00 for Windows (GraphPad Software, La Jolla, CA, USA) was used for statistical analysis. First, the D'Agostino-Pearson normality test was used to determine whether to perform parametric or non-parametric tests. One-way ANOVA test was performed to determine whether there are any statistically significant differences between the mean values of the controls and different asthma groups for the gene expression and protein levels. The same software was used to examine the correlations between the different parameters using Pearson correlation test in GraphPad Prism. Student *t*-test was used to look for the difference between two groups under a given experiment or treatment. A *p* < 0.05 is considered to be statistically significant.

## Results

### Severe Asthmatic Patients PBMCs Express Less AREG mRNA but Higher Protein Compared to Non-severe Asthmatics

**Figure 1:**
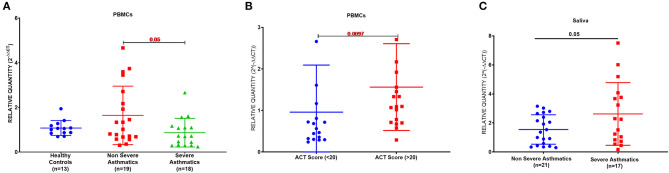
AREG mRNA expression as measured by RT qPCR in PBMCs and saliva of cohort of asthmatic vs. healthy control. **(A)** AREG mRNA level in PBMCs of the locally recruited cohort (cohort 1), healthy controls (*n* = 13), non-severe asthmatics (*n* = 20), and severe asthmatic (*n* = 18). **(B)** AREG mRNA gene expression in PBMC of a locally recruited cohort divided according to the ACT score into two groups: a well-controlled group (ACT > 20) and those with poor control (ACT < 20). **(C)** AREG mRNA gene expression in the saliva of independent validation locally recruited cohort (cohort 2), which includes non-severe asthmatics (*n* = 21) and severe asthmatic who are not on biological treatment (*n* = 17).

PBMCs AREG mRNA was lower in severe asthmatics compared to non-severe asthmatics (*p* = 0.05), (Figure 1A). PBMCs AREG mRNA correlated positively with IL17A plasma level (0.43, *p* < 0.05). Also, the expression correlated negatively with age (−0.38, *p* < 0.05), the use of oral corticosteroids (−0.58, *p* < 0.05), the number of exacerbations per year (−0.39, *p* < 0.05), and atopic status (−0.40, *p* < 0.05).

### AREG Was Higher in Well-Controlled Asthma

Asthma Control Test (ACT) is a patient self-administered tool for identifying those with poorly controlled asthma. The scores range from 5 (poor control of asthma) to 25 (complete control of asthma), with higher scores reflecting greater asthma control. An ACT score > 20 indicates well-controlled asthma. When we divided our patients accordingly, AREG mRNA expression in PBMC correlated positively with higher ACT scores, as shown in Figure 1B.

### AREG mRNA Was Higher in the Saliva of Severe Asthmatics Compared to Non-severe Asthmatics

A matched cohort of severe asthmatics (*n* = 17) vs. non-severe asthmatics (*n* = 21) was recruited for fresh saliva collections and RNA was extracted to detect AREG mRNA in the saliva. AREG showed significant upregulation in severe asthmatics compared to non-severe asthmatics, as shown in Figure 1C.

### Asthmatic Patients' Plasma and Saliva AREG Protein Level Is Higher Than Healthy Controls

**Figure 2:**
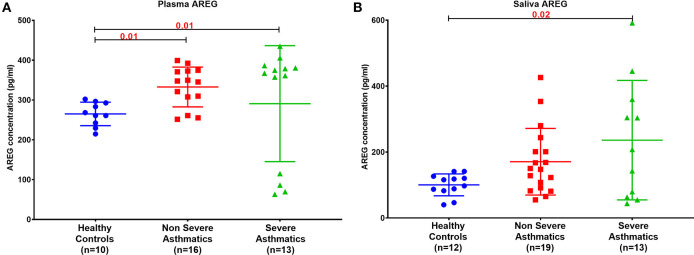
AREG protein level in **(A)** plasma and **(B)** saliva of the locally recruited cohort (cohort 1), including healthy controls, non-severe and severe asthmatic patients.

Plasma AREG level, as measured by ELISA, was higher in non-severe and severe asthmatic patients (333.24 ± 133.14 pg/mL, *p* < 0.01) compared to healthy controls (265.05 ± 29.49 pg/mL) as shown in Figure 2A. Interestingly, the saliva AREG level was also higher in severe asthmatic patients (247.35 ± 271.4 pg/mL, *p* = 0.02), as shown in Figure 2B. Indeed, plasma AREG correlated positively to saliva AREG level (*r* = 0.3671, *p* = 0.02), indicating the feasibility of using saliva to reflect the plasma level of AREG. AREG plasma level correlated negatively with PBMC AREG mRNA expression (*r* = −0.37, *p* < 0.05).

### Blood and Saliva AREG Protein Levels Correlate With Patient's Atopy Status and Allergic Rhinitis

**Figure 3:**
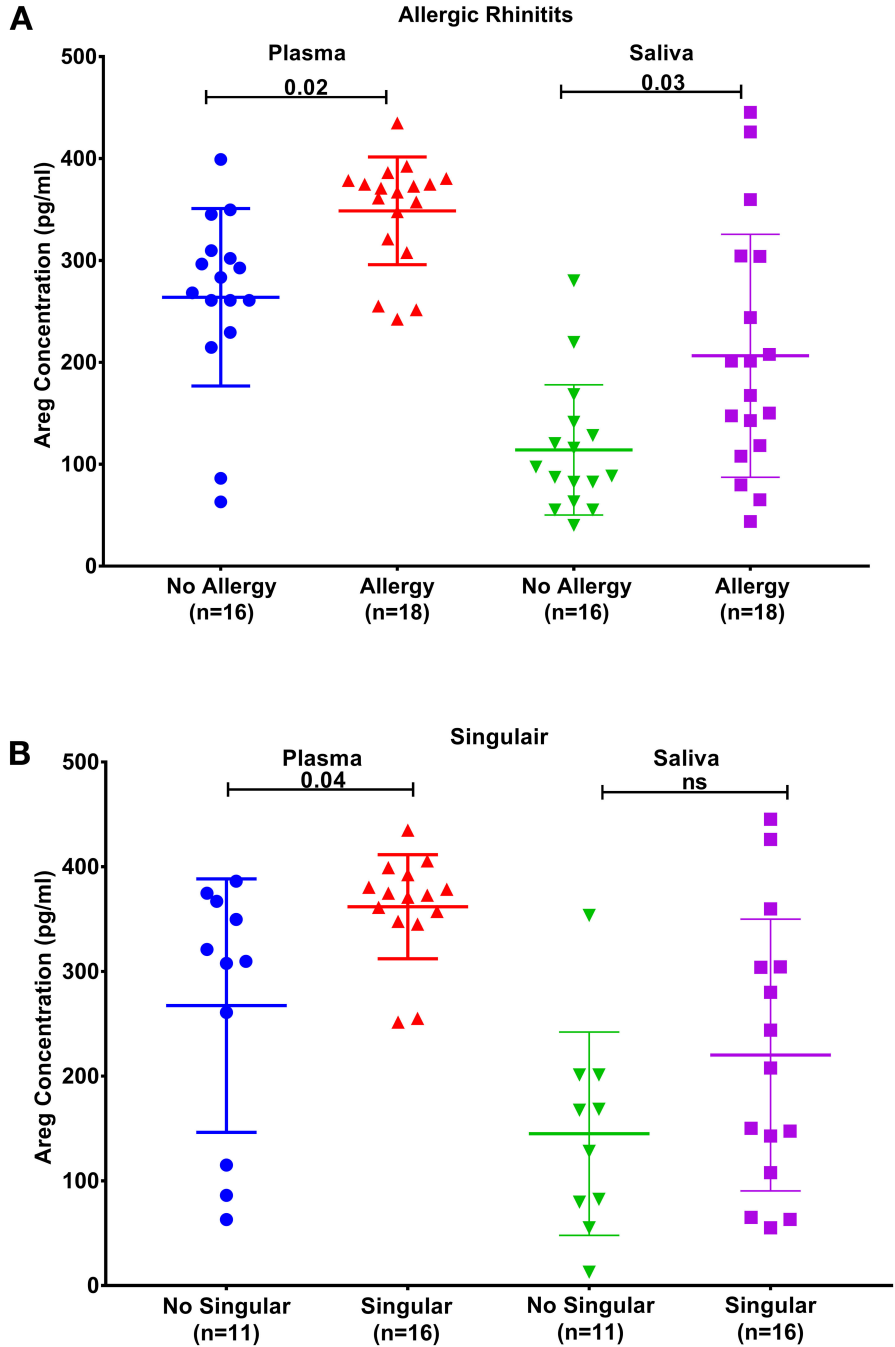
AREG protein levels in plasma and saliva. **(A)** in asthmatic with allergic rhinitits compared to those who are not and **(B)** in asthmatics currently on Montelukast sodium (Singulair) compared to those who are not on Singulair.

Plasma AREG levels had a positive correlation with ACT (*r* = 0.32, *p* < 0.05), allergic rhinitis status (*r* = 0.35, *p* = 0.03, Figure 3A), atopy status (*r* = 0.35, *p* = 0.03), neutrophil percentage in patients' blood (*r* = 0.45, *p* = 0.01), and the use of Montelukast sodium (Singulair) (*r* = 0.39, *p* = 0.04, Figure 3B). Interestingly, plasma AREG levels correlated positively with plasma POSTN levels (*r* = 0.34, *p* = 0.03). Correlation between Plasma AREG concentrations, and the cohort demographics and laboratory tests are listed in Table 4.

**Table 4:** Correlation between plasma AREG concentrations, and the cohort demographics and laboratory tests.

**AREG plasma (pg/mL)**	**vs. AREG saliva (pg/mL)**	**vs. Atopy status**	**vs. Allergic rhinitis status**	**vs. Blood neutrophil (%)**	**vs. POSTN plasma (pg/mL)**
Pearson correlation test	0.3671	0.3547	0.351	0.4494	0.3436
*r*					
95% confidence interval	0.04391–0.6207	0.02475–0.615	0.02544–0.6092	0.07192–0.7143	0.01704–0.6039
*P* (two-tailed)	0.0234	0.0312	0.0307	0.0187	0.0347
Number of XY pairs	38	37	38	27	38

On the other hand, saliva AREG levels correlated significantly with key clinical indicators for asthma assessment compared to plasma AREG, as listed in Table 5. Beside allergic rhinitis (*r* = 0.42, *p* = 0.02) and atopy status (*r* = 0.37, *p* = 0.02), saliva AREG level correlated positively with ACT score (*r* = 0.38, *p* = 0.04), FEV1 (% predicted) (*r* = 0.47, *p* = 0.001), FEV1/FVC (*r* = 0.48, *p* = 0.02) and eczema status (*r* = 0.35, *p* = 0.03). Interestingly, saliva AREG level correlated positively with the age of onset as adult asthmatics showed higher levels (*r* = 0.31, *p* < 0.05).

**Table 5:** Correlation between saliva AREG concentrations, and the cohort demographics and laboratory tests.

**AREG saliva (pg/mL)**	**vs. ACT score**	**vs. Atopy status**	**vs. Allergic rhinitis status**	**vs. Eczema status**	**vs. FEV1 (% predicted)**	**vs. FEV1/FVC**
Pearson correlation test	0.3895	0.3744	0.4216	0.3554	0.6458	0.487
*r*						
95% confidence interval	−0.009556–0.6815	0.04223–0.632	0.1032–0.6616	0.02557–0.6155	0.2959–0.8429	0.06913–0.7595
*P* (two-tailed)	0.0492	0.0245	0.0094	0.0309	0.0012	0.0215
Number of XY pairs	26	36	37	37	22	22

### AREG Protein Expression Was Higher in Bronchial Epithelial Cells of Mild Asthmatics Compared to Those of Moderate and Severe Patients as Well as Healthy Controls

**Figure 4:**
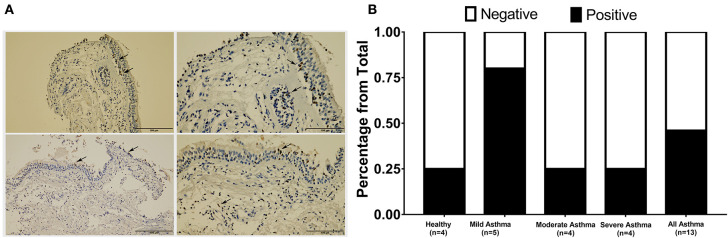
Immunohistochemical staining of AREG in bronchial biopsies of healthy, non-severe (mild and moderate) and severe asthmatic patients. **(A)** Arrows showing positively stained epithelial cells, **(B)** percentage of positive AREG IHC staining sample among all samples examined in each group.

AREG expression in lung tissue and its cellular localization were investigated using bronchial biopsies from asthmatic patients (*n* = 13) and healthy controls (*n* = 4), as shown in Figure 4. The asthmatic samples were from mild (*n* = 5), moderate (*n* = 4), and severe (*n* = 4) patients. Immunohistochemical expression of AREG was high in bronchial epithelial pseudostratified cells, while lower expression was observed in fibroblasts and other cells, including infiltrating immune cells. Interestingly, AREG expression in a healthy epithelium was nuclear while it was cytoplasmic in asthmatic samples. As expected, AREG expression was positive (6 out of 13, 46.15%) in asthmatic patient's samples compared to only 1 out of 4 samples in the control group (25%). Interestingly, AREG expression was positive in 4 out of the five mild asthmatic samples (80%) compared to only 1 case out of the four moderate (25%) and severe asthmatic samples (25%), *p* = 0.0495.

### AREG mRNA Level Was Higher in Non-severe Asthmatic Bronchial Epithelial Cells Compared to Healthy Controls and Severe Asthmatic

**Figure 5:**
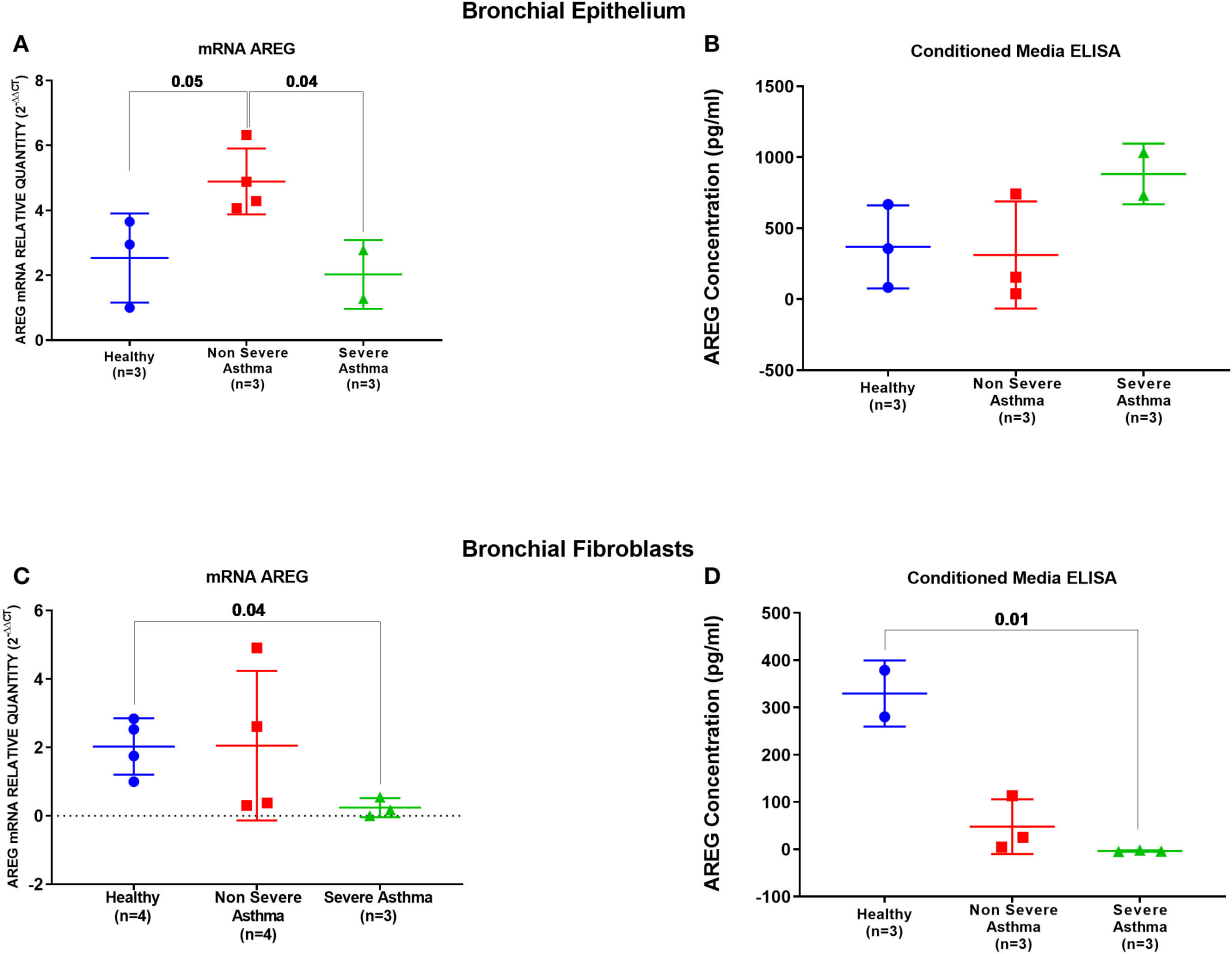
AREG mRNA and secreted protein in bronchial epithelium cells and fibroblasts of healthy, non-severe, and severe asthmatic individuals. Assessment of AREG in asthmatic bronchial epithelium compared to the healthy epithelium on the **(A)** mRNA level measured by qRT-PCR, **(B)** secreted AREG protein levels by ELISA. Similarly, AREG was assessed in asthmatic fibroblasts compared to the healthy fibroblasts for the **(C)** AREG mRNA levels by qRT-PCR and **(D)** secreted AREG protein levels by ELISA.

AREG mRNA and protein levels were measured in primary bronchial epithelial cells and fibroblasts from asthmatic patients and healthy controls. AREG mRNA expression was upregulated in non-severe asthmatic bronchial epithelial cells compared to healthy controls (*p* = 0.05), and severe asthmatics (*p* = 0.04), Figure 5A. Using ELISA, secreted AREG levels detected in the conditioned media of bronchial epithelium using ELISA showed no significant difference between asthmatics and healthy controls, as shown in Figure 5B. Nevertheless, asthmatic bronchial epithelial cells secrete higher amounts (more than five times) of soluble AREG in their conditioned media compared to their asthma severity matching fibroblasts, indicating that epithelial cells are the primary source of pulmonary AREG in asthma. Moreover, bronchial fibroblasts of severe asthmatics (*n* = 3) showed lower expression of AREG mRNA compared to healthy fibroblasts (*n* = 4) (*p* = 0.04), which goes in line with the ELISA results (*p* = 0.01), Figures 5C,D.

### Asthmatic Fibroblasts Secrete More AREG Ectoderm Shedders ADAM8, ADAM9, ADAM10 Compared to Healthy Fibroblasts

**Figure 6:**
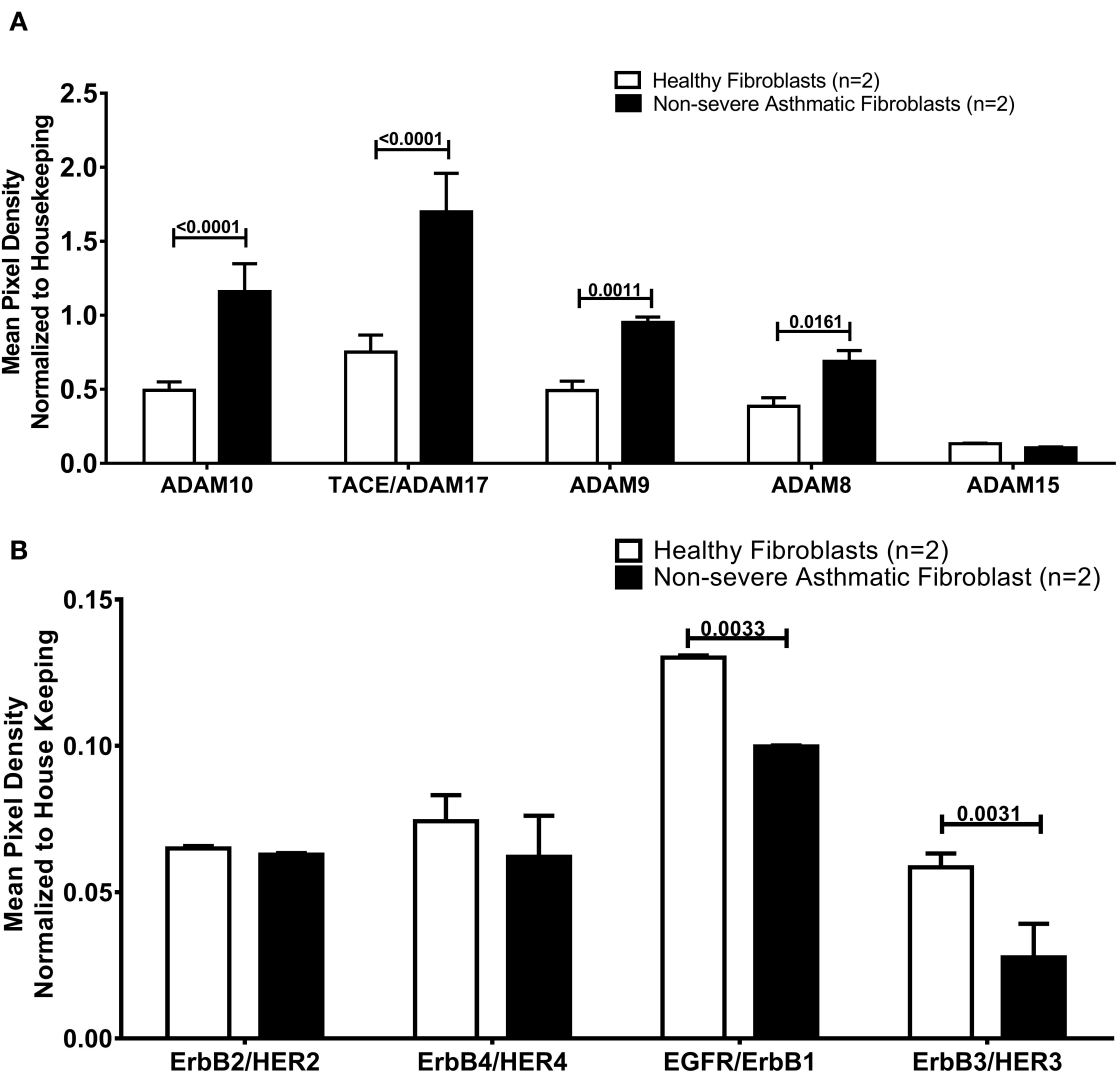
Levels of soluble receptors shed in the conditioned media of healthy and non-severe asthmatic fibroblasts using non-hematopoietic and the common analytes array. **(A)** Protein expression of AREG shredders: ADAM 8, 9, 10, 15, and 17 in non-severe asthmatic fibroblasts compared to healthy fibroblasts. **(B)** Protein expression of shed ErbBl-4/HER1-4 in non-severe asthmatic fibroblasts compared to healthy fibroblasts.

As the secreted levels of AREG matched its mRNA expression in severe asthmatic fibroblasts, we investigated the reason behind the low secreted levels of AREG in non-severe asthmatic fibroblasts despite of its high mRNA expression levels (Figure 6). We profiled the shredded proteins in their conditioned media and found that asthmatic fibroblasts secrete higher AREG ectoderm shedders ADAM8 (*p* = 0.01), ADAM9 (*p* = 0.001), ADAM10 (*p* < 0.0001) and ADAM17 (*p* < 0.0001) compared to healthy fibroblasts (Figure 6A). Another interesting finding is that non-severe asthmatic fibroblasts shed less ErbB1 (*p* = 0.003) and ErbB3 (*p* = 0.003) receptors compared to healthy fibroblasts (Figure 6B).

## *In Silico* Validation

### AREG mRNA Expression Was Significantly Different in Asthmatic Bronchial Epithelium, Fibroblasts Using Biopsy or Brush

AREG showed consistent differential expression in asthmatic lung epithelium and fibroblasts using biopsy or brush in publicly available bronchial epithelium datasets and confirmed *in vitro* (Table 6).

**Table 6:** Summary of *in silico* and *in vitro* analysis of AREG mRNA expression in different settings of asthma.

**Genes**	**Bronchial epithelium**		**Bronchial fibroblasts**		**Biopsy vs. brush**		**OS = Oral Steroids**	**Th2 high**	**Th2 low**
	***In silico***	***In vitro***	***In silico***	***In vitro***	**Healthy**	**Asthma**			
AREG	UP	UP	UP (the dataset available was with no details on severity)	Down in severe asthma	Both	-	-	-	-

### AREG mRNA Expression Was Significantly Different in Severe Asthmatic Compared to Severe Wheezing Children Only

**Figure 7:**
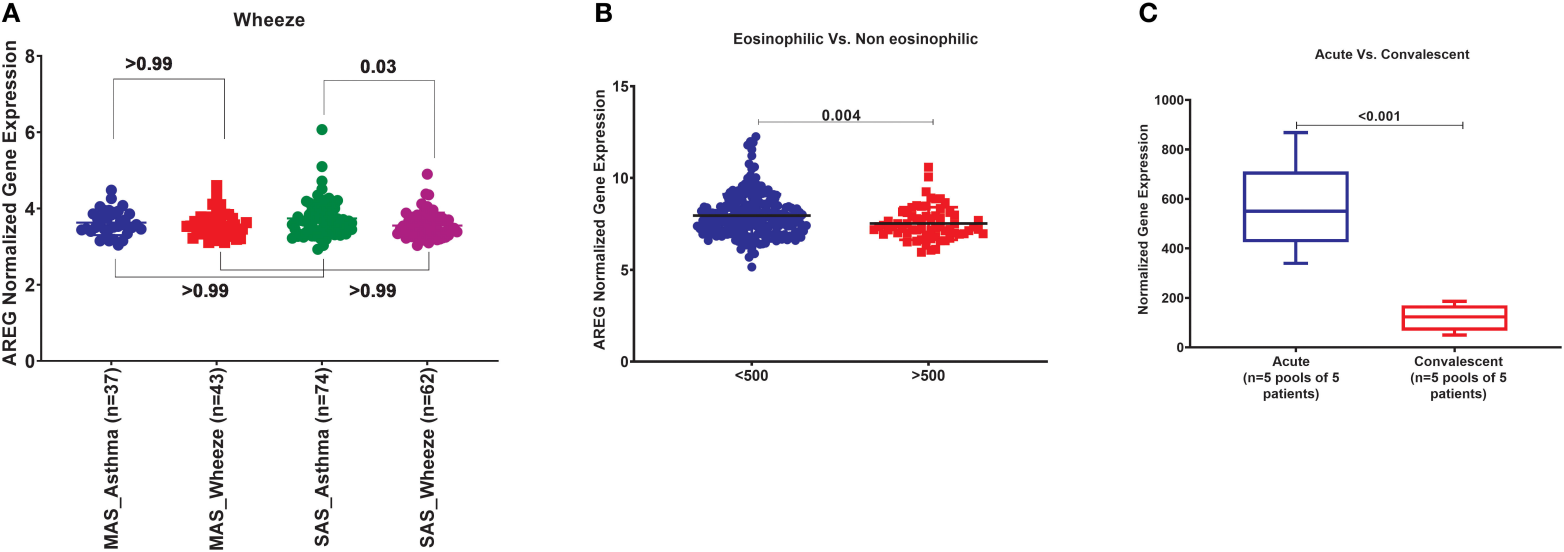
**(A)** AREG normalized gene expression in whole blood of children with non-severe and severe wheezes were compared to non-severe and severe asthmatics extracted from the expression profile of publically available datasets (GSE123750), where non-severe asthmatic (MAS_Asthma) and severe asthmatic (SAS_Asthma) patients were compared to patients presented with non-severe wheezes (MAS_Wheeze) and severe wheezes (SAS_Wheeze). **(B)** Normalized gene expression of genes identified in whole blood of eosinophilic asthma (>500 cells) compared to non-eosinophilic (eosinophils count less than 500) extracted from the expression profile of publicly available datasets (GSE137394). **(C)** Normalized Gene Expression of Genes Identified in PBMC5 taken from asthmatic patients during the acute/exacerbation phase and the convalescent phase extracted from the expression profile of publicly available datasets(6SE16032).

In order to explore the correlation of AREG mRNA in wheezing in asthmatic vs. non-asthmatic children, (GSE123750) gene set was explored. AREG mRNA expression in whole blood of children with non-severe and severe wheezes was compared to non-severe and severe asthmatics. As shown in Figure 7A, AREG mRNA expression was only significantly higher in severe asthmatic compared to severe wheezing children (*p* = 0.03). However, there was no significant difference in the gene expression of AREG between non-severe and severe asthmatic, indicating it is asthma specific markers.

### AREG mRNA Expression in Whole Blood of Eosinophilic Asthma Was Significantly Lower Compared to Non-eosinophilic

In order to explore the correlation of AREG mRNA to the eosinophilic phenotype of asthma, (GSE137394) gene set was, as shown in Figure 7B. AREG was significantly lower in eosinophilic asthmatics (*p* = 0.004). This indicates that AREG can identify a non-eosinophilic subtype of a severe asthma phenotype.

### PBMCs of Asthmatic Patients During Exacerbation Phase Express Higher AREG Compared to Convalescent Phase

GSE16032 dataset was chosen to identify the differential expression of the selected genes in PBMCs taken from asthmatic patients during the acute/exacerbation phase and the convalescent phase, as shown in Figure 7C. Interestingly, AREG was upregulated in the acute/exacerbation phase of asthma and returned back to the basal level when the attack subsides. This highlights the possible use of AREG as a marker for this specific disease phase.

## Summary in Blood

Since, PBMCs mRNA expression was associated with asthma control score and AREG saliva mRNA was upregulated in severe asthma compared to non-severe asthmatics, we decided to explore its dynamics to understand its role in initiating and progression of the disease. AREG mRNA in the blood of drug naïve asthmatics compared to healthy, can differentiate severe asthmatic wheezes from others, can differentiate eosinophilic from non-eosinophilic, can differentiate acute attacks from convalescent-phase, and its expression was not affected by viruses (Table 7).

**Table 7:** Summary of *in silico* analysis of the AREG genes in blood of asthmatic patients in different settings compared to healthy controls.

**Genes**	**Whole blood**				
	**Asthma**	**Asthma**	**Eosinophilic**	**Acute**	**Viral**
		**wheezes**			
AREG	–	UP	Down	UP	–

*AREG expression in whole blood of asthmatics in different phenotypes, in asthmatic with wheezes vs. non-asthmatics wheezers, in eosinophilic asthmatic vs. non-eosinophilic, in acute vs. convalescent (acute) and during viral infection*.

### AREG Has Never been Linked to SNPs in Asthma

Genome-wide association studies (GWASs) of asthma have identified many risk alleles and loci that can be a potential source for diagnostic biomarkers. NHGRI-EBI GWAS Catalog was explored to identify 1,174 SNPs with the strongest Asthma-related SNP-risk allele. Interestingly, none of these SNPs were reported in AREG.

### Severe Asthmatics Epithelium and Blood That Showed Higher AREG Expression Showed Different Immune Cells Populations Than Healthy Controls

**Figure 8:**
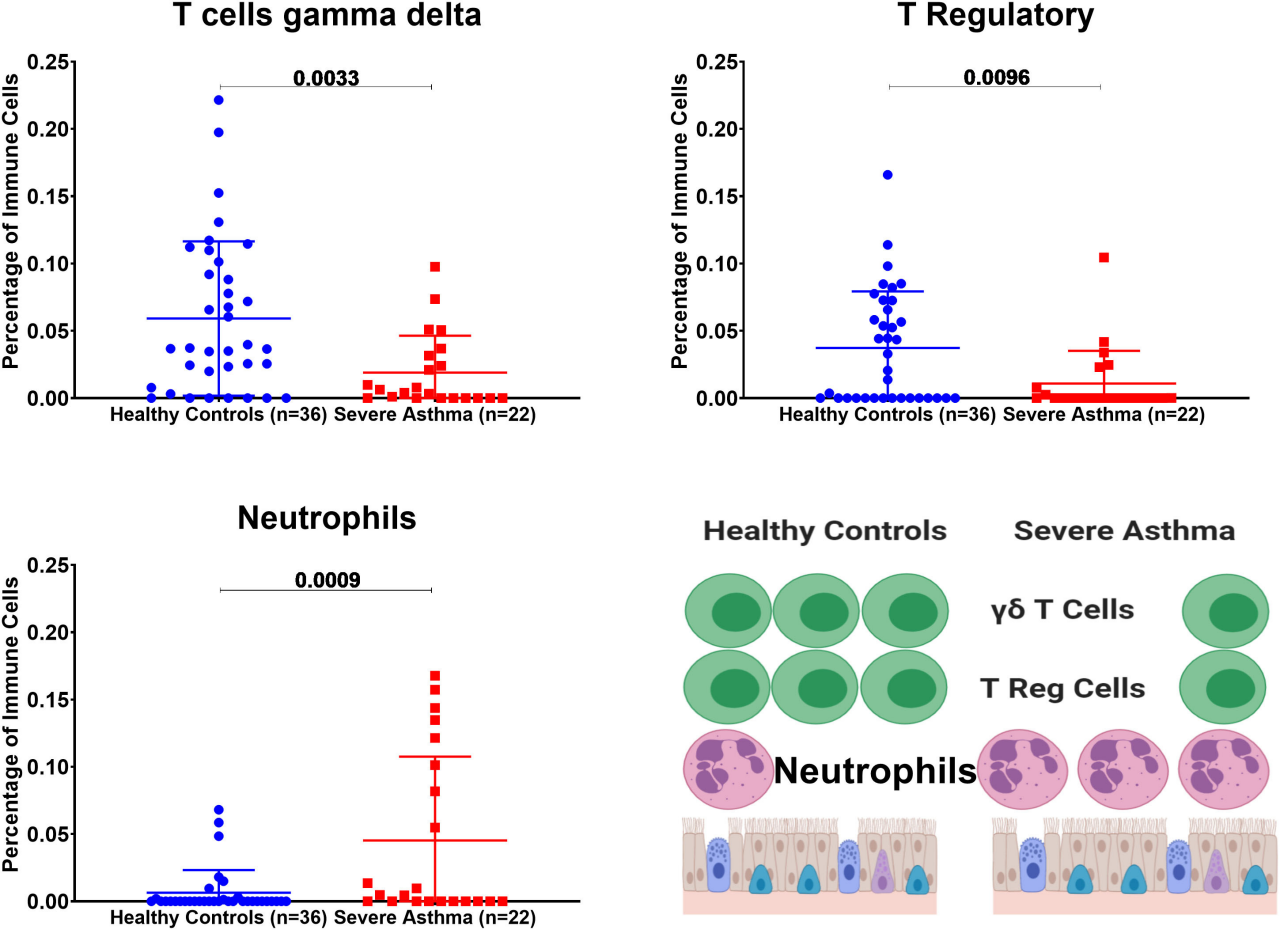
Estimating immune cells populations in the bronchial epithelium of (c3SE64913) dataset in healthy controls (*n* = 36) compared to severe asthmatics (*n* = 22). Bronchial epithelium genes expression was loaded to CIBERSORT *in silico* flow cytometry.

We investigated if immune cell populations are different between asthmatic and healthy controls using the transcriptomic expression of the bronchial epithelium to predict the cell type and their state of activation. CIBERSORT tool, a digital cytometry that can determine cell type abundance and expression from bulk tissues (40), was used for that purpose. We used CIBERSORT tool on Bronchial epithelium transcriptomic data of (GSE64913 and GSE67472) datasets and blood transcriptomic dataset (GSE69683) of a well-characterized and a large cohort of patients with different asthma severity compared to healthy controls to estimate the percentage of the immune cells (Figure 8).

### Bronchial Epithelium of Severe Asthmatic With Higher AREG mRNA Expression Showed a Lower Percentage of Gamma Delta (γδ) T and T Regulatory Cells but Higher Percentage of Neutrophils

In severe asthmatic bronchial epithelium with higher AREG mRNA expression, the percentage of infiltrating Gamma Delta (γδ) T and Regulatory T cells was lower than healthy controls, while there was an increase in the percentage of neutrophils (Figure 8).

### Peripheral Blood of Severe Asthmatics Showed a Higher Percentage of Eosinophils, Neutrophils and Activated NK Cells Compared to Healthy Controls

**Figure 9:**
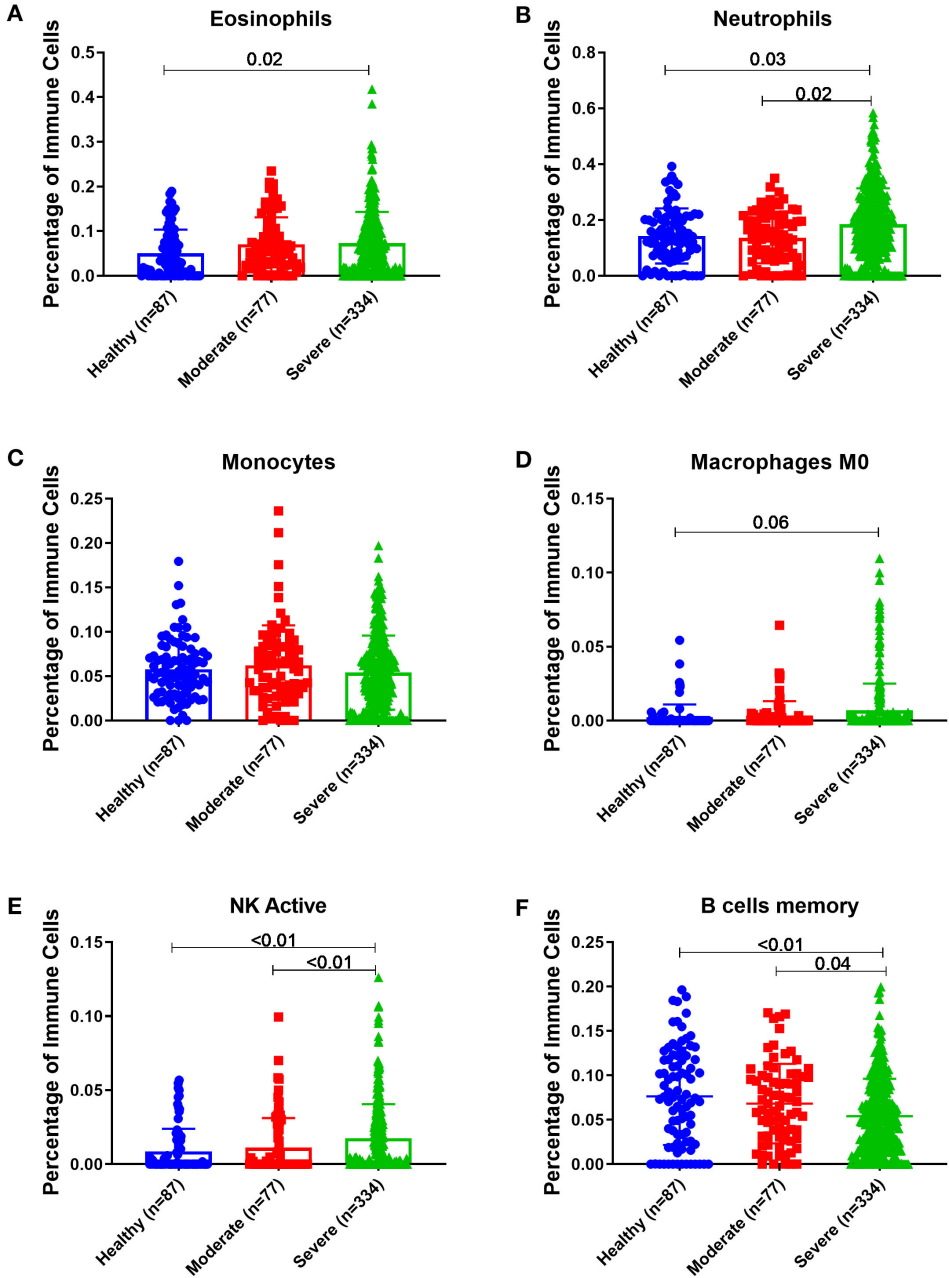
Estimating immune cells populations in blood of (GSE69683) dataset to compare healthy controls (*n* = 87), non-severe asthma (*n* = 77), and severe asthmatics (*n* = 334). Blood genes expression was loaded to CIBERSORT *in silico* flow cytometry and immune cells which showed significant differences between the groups are shown. **(A)** Eosinophils, **(B)** Neutrophils, **(C)** Monocytes, **(D)** Macrophages M0, **(E)** NK active, and **(F)** B cells memory.

Estimating the immune cells population in the blood of a large cohort of asthmatic patients with different severities compared to healthy controls using the transcriptomics data from (GSE69683) dataset revealed a similar trend of cells abundance of that in bronchial epithelium in terms of neutrophils and macrophages. As shown in Figure 9, eosinophilia was more evident in severe asthmatic blood than in local bronchial epithelium. Severe asthmatic patients showed a higher percentage of eosinophils in their blood than healthy controls (*p* = 0.02). However, the percentages of monocytes and macrophages percentages were not statistically different between the groups. Surprisingly, memory B cells showed a significant difference in severe asthmatics compared to non-severe asthmatics (*p* = 0.04) and healthy controls (*p* < 0.01).

Interestingly, peripheral neutrophilia and activated NK was consistent with airway findings. Blood neutrophils and activated NK percentages were higher in severe asthmatics compared to non-severe asthmatics and healthy controls (*p* < 0.05).

## Discussion

To our knowledge, no studies used saliva, while very few studies used blood levels to discover markers related to asthma pathogenesis. This is the first study to investigate AREG blood and saliva expression levels and associate them to asthma. Other studies focused on AREG expression in sputum (17), damaged bronchial epithelium (41), fibroblasts (42), smooth muscle cells (43, 44) from bronchial biopsies and hematopoietic cells such as eosinophils (19), and basophils (45) in asthma and allergic conditions.

As illustrated in Figures 1, 2, our results showed that AREG plasma and saliva levels were higher in asthmatic patients compared to healthy controls. AREG plasma level was significantly different between healthy and non-severe asthmatic (*p* < 0.0001).

Currently, in asthma research, there is a growing interest in replacing the available invasive bronchoscopy sampling and sputum induction with less invasive options like blood, urine, and exhaled gas biomarkers (4). These biomarkers can aid in a personalized and targeted approach to manage asthmatic patients, especially those with severe refractory asthma (46).

Our results confirmed that AREG level in plasma and saliva are simple, minimally invasive tools that can be used as biomarkers to assess asthma pathogenesis. Salivary marker measurements of various diseases is a new concept and currently is carried out mostly in head and neck cancers (47) and oral diseases (48). However, these assays are mostly DNA based. More recently, a predictive genetic assay measuring salivary mRNA was used to predict the onset of esophageal cancer (patent no: WO2017137427A1). As saliva sampling is non-invasive and is easy to collect, it represents an attractive tool for measuring asthma biomarkers (25).

This promising discriminating power of our results can be an additional bedside marker to help the managing physician in diagnosing early stages of asthma with borderline clinical criteria. As for plasma, AREG saliva level was not able to differentiate between different asthma severity indicating its association with asthma as a disease; this might be explained by the limitation of the sample size and the effect of aggressive asthma medication on AREG levels in severe asthmatics.

Determining the presence of eosinophilic inflammation, or high Th2 cytokines phenotype is essential for patient stratification for targeted asthma therapy (49). Currently, peripheral blood eosinophil count is considered as a surrogate marker for eosinophilic inflammation of the airways (50); hence, finding a new reliable biomarker to predict eosinophilic asthma is needed (4). Therefore, correlating AREG expression with other pathological parameters, specifically those related to eosinophilic inflammation or high Th2 cytokines phenotype is essential.

Our results showed that AREG protein level in asthmatic patients' plasma and saliva correlate with clinical allergic phenotypes such as allergic rhinitis and atopy. Patients with a history of allergic rhinitis showed specifically a higher plasma AREG level compared to those with no history of allergic rhinitis. Allergic rhinitis is a common comorbidity of asthma and contributes to asthma severity (51) and poor asthma control (52). Asthma and allergic rhinitis share similar local and systemic eosinophil inflammation (53). AREG was shown to be secreted by eosinophils to participate in some physiological and pathological conditions *in vivo* (19). Asthma specific memory Th2 cells were shown to induce AREG to reprogram eosinophils to produce osteopontin leading to enhanced airway fibrosis (20).

On the other hand, AREG protein level in plasma correlates with the level of periostin, a known marker for Th2 high asthma phenotype (54), which can predict airway eosinophilia in patients with severe asthma (55). Periostin was found to be involved in atopic conditions such as dermatitis, rhinitis/rhinosinusitis, allergic skin inflammations; and its upregulation was shown to induce extracellular matrix (ECM) tissue remodeling (56). Beside eosinophils and mast cells, increased levels of periostin have also been detected in relation to neutrophils (56). On the other hand, periostin, was shown to play a vital role in chemokines induction to recruit neutrophils and macrophages that participate in pulmonary fibrosis in mouse model (57). Positive correlation between AREG and periostin might augment the role of neutrophils in non-eosinophilic severe asthma.

Of note, plasma AREG levels correlated positively with neutrophil levels in patients' blood. It is known that neutrophilia correlates with asthma that is refractory to the mainstay of asthma treatment (corticosteroids) (58). It was shown that during epithelial damage and repair, neutrophils enhance AREG production by epithelial cells to promote tissue repair (59). It can be suggested that patients with high circulating AREG might be in need of that level to be used for the massive tissue repair process ongoing during asthma.

A non-allergic asthmatic is a non-sensitized individual who can develop eosinophilic inflammation without exposure to an allergen through the activation of ILC2 cells that do not need allergen-specific IgE (22). Non-allergic patients may have innate immunity or neutrophilic airway inflammation independent of Th2 cells (27). Triggers of asthma in non-allergic patients can vary from external triggers like viral infections, cigarette smoke, diesel particles, and ozone to patient's intrinsic factors like stress, exercise, and obesity (1).

The type and quantity of inflammatory cells in the airway of asthma is an attractive research area now as it can guide the selection of biological treatment (60). Differentiating neutrophilic asthma (usually characterized by acute severe asthma exacerbations and steroid-resistant) from the eosinophilic phenotype is very important as the later phenotype can respond to corticosteroids and targeted therapies (61).

During acute lung injury, AREG was shown to suppress ICAM-1 expression and neutrophil accumulation (62). However, if neutrophils infiltration occurs, their elastase can promote myofibroblast differentiation that ends up with lung fibrosis; thus, AREG can limit fibrosis by limiting neutrophil arrival earlier in inflammation (63). This model is opposite to the skin model of psoriasis, where AREG enhanced the transmigration of human neutrophils through epithelial cell monolayers (64).

Our results showed that in severe asthmatic bronchial epithelium, the percentage of infiltrating Gamma Delta (γδ) T and Regulatory T cells was lower than in those of healthy controls. The high content of neutrophils in severe asthmatic bronchial epithelium compared to healthy controls matches the decrease in γδ T and Tregs as it might indicate more inflammatory consequences of lack of the primary tolerogenic and regulators of an immune response.

Estimating the immune cells population in the blood of a large cohort of asthmatic patients with different severities compared to healthy controls revealed a similar trend of cells abundance of that in bronchial epithelium in terms of neutrophils and macrophages. Eosinophilia was more evident in severe asthmatic blood than in local bronchial epithelium.

Interestingly, peripheral neutrophilia was consistent with airway neutrophilia. Increased local airway neutrophils count in asthma in neutrophilic asthma is associated with steroid resistance, suggesting that the neutrophil plays a role in asthma pathophysiology (65). Increased airway neutrophils in severe asthma and Th2 low phenotype might indicate specific endotype of asthma that share neutrophilia and eosinophilia but not Th2-high phenotype. In response to allergens, activation of LPS receptor TLR4 on lung epithelial cells can stimulate CXCL8 chemokine secretion to recruit neutrophil to the lung in association with increased eosinophilic inflammation, IgE, Th2 cytokines and mucus secretion (66). TLR signaling activation during the sensitization phase recruit neutrophils and inflammatory monocytes, to attenuate allergic symptoms by promoting the Th1-associated cytokines (67).

High levels of IL17 are linked to both eosinophils and neutrophils infiltrates in the airways of severe asthmatics (68). In neutrophilic asthma, Th17 cells released interleukins can recruit macrophages, and lymphocytes alongside neutrophils (69). Despite being glucocorticoid sensitive in some autoimmune diseases, Th17 cells are glucocorticoid resistant in asthma (70).

During allergen exposure, the first immune cells arriving the airways are the neutrophils that usually have a short life before it undergoes apoptosis, except in severe asthmatics who showed defective neutrophil apoptosis and/or clearance (71).

Another interesting finding was the correlation between plasma AREG level and the use of Montelukast sodium (Singulair); a selective and orally active leukotriene receptor antagonist that inhibits the cysteinyl leukotriene CysLT1 receptor (72). Our results showed that asthmatic patients who are on regular Singulair treatment have a higher level of AREG in their plasma compared to those who are not using Singulair. CysLTs as a potent contractile agonist of airways smooth muscles can stimulate their amphiregulin release and enhance their proliferation (73). Moreover, cysteinyl leukotriene was recently found to activate human ILC2s to secrete amphiregulin (72). This can have an important translational impact on patients' stratifications and appropriate drug selection.

Immunohistochemistry staining of bronchial biopsies showed that AREG staining intensity was higher in non-severe mild asthmatic epithelium when compared to healthy controls (Figure 4). These findings confirm the role of AREG in the sequence of events in asthma pathogenesis involving both bronchial epithelial cells and fibroblasts. Our results agree with previous studies where AREG level in sputum was associated with asthma severity (17) and was found to be increased in airways during an acute asthma attack (18).

Our results confirmed that AREG is locally upregulated in the asthmatic lung. AREG is constitutively expressed during development and physiologic processes but rapidly upregulated in damaged epithelium to restore tissue integrity (15). Asthmatic bronchial epithelial cells had higher AREG mRNA expression and protein levels than healthy ones but no significant difference in the secreted levels. However, AREG secretion in conditioned media was higher in bronchial epithelial cells compared to their asthma severity matching bronchial fibroblasts confirming that epithelial cells are the primary source of local lung cells in asthma (Figure 5). The insignificant difference between AREG secreted levels of healthy and asthmatic epithelial cells despite the overexpression of its mRNA levels can be explained by the complex regulatory network controlling AREG synthesis and secretion.

AREG was reported to show the ability to restore tissue integrity (15), through the mechanism of shedding to bind to autocrine or paracrine EGF receptor family. AREG ectodomain shedding is mediated by ADAM17 (74) and under some conditions ADAM8 and ADAM15 that are called sheddases (75). Most of these enzymes are secreted proteins (76). This processing adds another layer of regulation of AREG (77). Our results showed that asthmatic fibroblasts showed higher shedding of AREG ectoderm shedders (ADAM8, 9, 10, and 17). This goes with previous reports that in asthmatics airways, ADAMs, and ADAMTSs and their inhibitors are involved in asthma development (76).

As illustrated in Figure 6A, asthmatic fibroblasts showed higher shedding of AREG ectoderm shedders (ADAM8, 9, 10, and 17). The loss of the shedders can decrease the amount of the shed protein in the conditioned media. These shedders can still act on the adjacent cells to shed more AREG and increase its soluble forms. ADAM8 plays a proinflammatory role in asthma airway inflammation, and ADAM8-deficient mice failed to develop an experimental model of asthma (78). Nevertheless, there is a controversy about its function in asthma, ADAM8 can induce or inhibit the transmigration of leukocytes in airway inflammation of asthma (79). Also, ADAM8 was shown to mediate the infiltration of eosinophils and Th2 lymphocytes to airways of asthmatic patients (80). On the other hand, some reports showed that ADAM8 facilitates neutrophil migration to the airways in severe asthma and COPD (81).

Moreover, as illustrated in Figure 6B, our results revealed that non-severe asthmatic fibroblasts shed less ErbB1 and ErbB3 EGFR receptors compared to healthy ones. The indispensability of ErbB1/EGFR for AREG-mediated responses was previously reported in lung structural cells, and it was suggested that ErbB1-ErbB3 and/or ErbB2-ErbB3 heterodimers are involved in the AREG-mediated responses (82). AREG soluble form can act in an autocrine fashion to increase the stability and accumulation of its active EGFR (83). Alternatively, it was recently documented that in stressed cells (like the case of asthma), mRNA concentrations can transiently escalate in pulse-like pattern and return to basal level, while protein concentrations establish a new steady-state (84). It was reported that upon stimulation, cells would release soluble ADAM17 in exosomes to reach more distant substrates or cells (85).

AREG can activate intracellular signaling network that controls cell survival, proliferation, and motility (13). AREG binding to EGFR induced EGFR homodimerization or heterodimerization with ErbB2, ErbB3, and ErbB4 to trigger intracellular signals (13). The membrane proteoglycan, CD44, is a cofactor for the interaction of AREG with EGFR (86).

In contrast to most of EGFR ligands, AREG did not generate nuclear translocation of EGFR but showed markedly different patterns of EGFR internalization and trafficking (87). The ubiquitination of pro-AREG accelerated its half-life on the cell surface with the subsequent trafficking to intracellular organelles (88). The remaining cytoplasmic carboxy-terminal domain of AREG plays a vital role in regulating autocrine growth through the EGFR (77). The released AREG can circulate in a recently discovered type of extracellular nanoparticle called exomeres can modulate EGFR trafficking and prolong EGFR downstream signaling in recipient cells (89). Cells lacking AREG showed growth arrest that is restored by proAREG and AREG-CTD (90). AREG has “geographically” specific and unique intracellular signaling pathways that can determine whether cells grow or differentiate (77).

Helminth-induced type 2 inflammation enhance EGFR expression and AREG production under the effect of IL33 cytokine; later, AREG-EGFR activation will stimulate IL13 secretion needed for host resistance (15, 91). The upregulated AREG can reprogram infiltrating eosinophils to an inflammatory phenotype that produces an excess of profibrotic immunomodulatory protein osteopontin (20). IL5 stimulated eosinophils can contribute to the progression of airway remodeling, through the production of AREG (19).

There is translational evidence indicating upregulated expression of EGFR appears in the airways of asthmatics and activity of this signaling pathway is enhanced in relation to asthma severity (92). Specifically mucus hypersecretion is induced by EGFR activation to promote goblet-cell metaplasia in severe asthma (12). EGFR signaling of the asthmatic epithelium has been reported to be increased in relation to disease severity through the capacity of driving neutrophil fate and function through elaboration of neutrophil-specific factors in relation to disease severity (28). Others have reported a strong correlation between EGFR expression and neutrophilic-specific chemokines in the epithelium of patients with severe asthma (93, 94).

The high AREG expression in blood and saliva of asthmatic patients can be linked to the local increase in AREG expression secreted by structural cells into the extracellular spaces. AREG will then enter the systemic circulation due to the increased microvascular permeability during tissue inflammation (95, 96). The increased AREG blood levels will most likely diffuse into saliva and hence used as a reflective marker for upregulated plasma levels (31).

One limitation of our study is the small size of the participants, although we tried to include all patients who fulfill the criteria, many of the patients refused to participate in the study. Since asthma is a heterogeneous disease, having larger cohort can be more informative about the applicability of the new biomarkers. The number of females in each group was higher than males, previous reports showed that mice males differentially produce and utilize AREG in their lungs in response to viral infections, with greater EGFR internalization and was linked to the combinational effect of testosterone and AREG, that can improve the repair and recovery of damaged tissue in males compared with females (97). On the other hand circulating AREG was undetectable with no significant variation during the menstrual cycle (98). On the other hand, AREG mRNA expression was peaked in the pregnant females and correlated positively with number of good-quality embryos under the induction by diverse luteinizing hormones (99).

In conclusion, our findings suggest circulating AREG expression level can be a reliable, non-invasive, and cost-effective biomarker that can provide additional discriminating power to the available clinical and laboratory tests of asthma.

## Data Availability Statement

All datasets generated for this study are included in the article/Supplementary Material.

## Ethics Statement

The studies involving human participants were reviewed and approved by the Ethics Committee of Dubai Health Authority and the University of Sharjah approved the study with REC (Research Ethics Committee) approval number DSREC-11/2017_04, and each subject gave written informed consent after a thorough explanation by the treating physician and the researchers. The patients/participants provided their written informed consent to participate in this study.

## Author Contributions

MH, RHam, and QH: conception and design of the study, data and sample collection, analysis and interpretation of data, and writing the manuscript. BM and RHam: conception and design of the study, clinical samples selections, and collections. MH and IH: *in silico* validation and analysis. SA, IH, and RHal: reviewing the manuscript. RO: primary lung cell lines and bronchial biopsies provision. NE, MH, and RHam: data collection and interpretation of ELISA and reviewing the manuscript. RR and MH: sample collections and cell lines maintenance. TV and LS: samples and data collections. All authors contributed to the article and approved the submitted version.

## Conflict of Interest

The authors declare that the research was conducted in the absence of any commercial or financial relationships that could be construed as a potential conflict of interest.

## Abbreviations

ACT: asthma control test
ADAM: a disintegrin and metalloproteases
AHR: airway hyperresponsiveness
AREG: amphiregulin
COPD: chronic obstructive pulmonary disease
DMEM: Dulbecco's Modified Eagle's medium
EDTA: ethylenediaminetetraacetic acid
EGF: epidermal growth factor
EGFR: the epidermal growth factor receptor
ELISA: enzyme-linked immunoassay
ErbB: erythroblastic leukemia viral oncogene
FBS: fetal bovine serum
Feno: fraction of exhaled nitric oxide
GEO: Gene Expression Omnibus
HDM: house dust mite
IgE: immunoglobulin E
IL17A: Interleukin 17 A
mRNA: Messenger RNA
PBMC: peripheral blood mononuclear cells
qRT-PCR: Real-Time qRT-PCR
ROC: Receiver Operating Characteristic curve
TH2: T helper cell type 2
TNFα: TNF-alpha.
